# 

**Published:** 1821-09-01

**Authors:** 


### BIBLIOGRAPHICAL RECORD;

OR

*Works received for Review, within the Quarter.*

1. Principes Generaux de Physiologie-Pathologique, coordonnés d'apres la doctrine de M. Broussais. Par L. J. Begin, Chirurgien Aide-Major a l'Hopital Militaire d'Instruction, &c. 1 tom. 8vo, pp. 390, 1820.

*Through Dr. Breschet.*

---

2. Refutation des Objections, Faites, a la Nouvelle Doctrine des Fievres; ou de la Non-existence des Fievres Essentiales. Par Louis-Charles Roche, M.D. One vol. 8vo, pp. 168. Paris, 1821.

---

3. Journal General de Medecine, for December 1820, and January, February, and March, 1821, in exchange for *Medico-Chirurgical Review.*

---

4. A Physiological Essay on the Sensibility of Animals; being the first part of a Series of Enquiries on the Nature of Life. By G—— W——. Octavo, sewed, pp. 40. London, 1820.

☞ *This little pamphlet contains many ingenious views and sensible reflections on several physiological subjects. We cordially agree with the author in the following sentiment, which concludes the pamphlet; "An apparent correct idea of death may be drawn from this theory of life, by considering it a loss of relation between a soul and the laws of this natural world. And all reasoning upon the soul's immortality may be suffered to rest on the admission of its indestructible nature, with reliance upon the Supreme Power for a re-establishment of a relation between it and the laws of some other, it is to be hoped, happy abode."* 39.

---

5. Journal de Physiologie Experimentale. Par F. Magendie, M. D. No. 2, April, and No. 3, July, 1821.

---

6. The New England Journal of Medicine and Surgery, and collateral Branches of Science. Conducted by a number of Physicians. Nos. 1 and 2 of vol. x. for January and April, 1821.

---

7. Practical Remarks on disordered States of the Cerebral Structures, occurring in infants. By Whitlock Nicholl, M. D. M. R. I. A. F. L. S. &c. Small octavo, pp. 94. London, 1821.

---

8. The History of Plague, as it has lately appeared in the Islands of Malta, Gozo, Corfu, Cephalonia, &c. detailing important facts, illustrative of the specific Contagion of that Disease, with particulars of the means adopted for its eradication. By J. D. Tully, Esq. Surgeon to the Forces, Member of the Ionian Academy, late Inspector of Quarantine, and President of the Board of Health of the Ionian Islands. One vol. 8vo, pp. 292. London, 1821.

☞ *The author, who has resided many years in plague countries, and was an eye witness of the disease, and of the salutary regulations put in force to check it, has adduced the most incontrovertible evidence of the specific contagion of plague. The work should be in the hands of all medical and other officers about to visit those countries where the disease is prevalent, and to these in particular we recommend it. We hope soon to exhibit an analytical view of its contents in this Journal.*

9. A Supplement to the Pharmacopœia: being a Treatise on Pharmacology in general; including not only the drugs and compounds which are used by practitioners of medicine, but also those which are sold by chemists, druggists, and herbalists, for other purposes; together with a collection of the most useful medical formulæ, an explanation of the contractions used by physicians and druggists; the medical arrangement of the articles of the London Pharmacopœia, with their doses at one view; a similar list of the Indigenous Plants of the British Islands, which are capable of being used in medicine, &c. also a very copious Index, English and Latin, of the various names by which the articles have been known at different periods. *A new and improved edition, considerably enlarged.* By SAMUEL FREDERICK GRAY, Lecturer on the Materia, Pharmaceutical Chemistry, and Botany. One closely printed volume, octavo, pp. 600. London, 1821.

☞ *The rapid sale of the former editions proves the sanction of the public, and the increase of matter in the present, must render it still most acceptable to the profession.*

---

10. A Conspectus of the Pharmacopœias of the London, Edinburgh, and Dublin Colleges of Physicians; being a practical Compendium of Materia Medica and Pharmacy. By A. T. THOMSON, F. L. S. &c. &c. &c. One vol. duodecimo, pp. 168. London, 1820.

☞ *This is the most useful vade-mecum for the pocket or desk of practitioners of all ranks, that exists in the English or any other language.*

---

11. Observations on some of the general Principles and on the particular Nature and Treatment of the different Species of Inflammation; being, with additions, the substance of an Essay to which the Jacksonian Prize, for the year 1818, was adjudged by the Royal College of Surgeons. By J. H. JAMES, Surgeon to the Devon and Exeter Hospital, and Consulting Surgeon to the Exeter Dispensary. One vol. 8vo, pp. 328. London, 1821.

---

12. Essays on Hypochondriasis, and other Nervous Affections. By JOHN REID, M. D. Member of the Royal College of Physicians, London; and late Physician to the Finsbury Dispensary. The *second* edition, with considerable additions. One volume, 8vo, pp. 440. London, June, 1821.

---

13. Practical Illustrations of Typhus Fever, &c. By JOHN ARMSTRONG, M. D. Republished in Philadelphia, with Notes, critical and explanatory, by Nathaniel Potter, M. D. Octavo, pp. 468. Philadelphia, 1821.

---

14. American Medical Recorder, Nos. 13 and 14, for Jan. and April, 1821.

---

15. [From the Publishers.] A Treatise on the Hydrocephalus Acutus, or Inflammatory Water in the Head. By L. A. GOLIS, of Vienna. Translated from the German, by ROBERT GOOCH, M. D. One vol. octavo, pp. 280. London, 1821.

---

16. A Treatise on the Nature and Treatment of Scrophula; describing its connexion with Diseases of the Spine, Joints, Eyes, Glands, &c. &c. founded on an Essay to which the Jacksonian Prize, for the year 1818, was adjudged by the Royal College of Surgeons. To which is added, a brief Account of the Ophthalmia, so long prevalent in Christ's Hospital. By EUSEBIUS ARTHUR LLOYD, Member of the Royal College of Surgeons in London, Senior Surgeon to the General Dispensary, Aldersgate Street, and late House-Surgeon to St. Bartholomew's Hospital. One vol. 8vo, 330 pages. London, 1821.

17. A Series of Lectures on the most approved Principles and Practice of Modern Surgery; principally derived from the Lectures delivered by Sir ASTLEY COOPER, Bt. F. R. S. &c. &c. at the united Hospitals of Guy and St. Thomas, and in which will be found some of the opinions of the most celebracted surgeons, from the time of Hunter to the present moment: interspersed with numerous cases. By CHARLES WILLIAM JONES. *Second Edition.* By CHARLES MINGAY SYDER, Surgeon. One vol. 8vo, pp. 448, small type. London, 1821.

☞ *This new edition is dedicated (by permission) to the eminent Baronet from whose lectures it was originally taken. It now comes, therefore, in a more authentic shape than before, and will undoubtedly attract considerable attention in the surgical world, as containing the opinions and practices of perhaps the first surgeon of the age in which we live. We have reason to believe that the original writer took down the ideas of Sir Astley Cooper's lectures, but not always correctly, and with little regard to order or language. Yet with these imperfections, the work will be found an invaluable repository of surgical knowledge.*

---

18. The Accoucheur's *Vade Mecum.* By JOHN HOPKINS, M. D. Physician Extraordinary to Her Royal Highness the Duchess of Kent's Household, and Physician-Accoucheur to the Westminster Lying-in Institution. In two volumes, duodecimo, 464 pages, *seventh edition.* London, 1820.

☞ *Of a* SEVENTH *edition it would be impertinent to obtrude an opinion. The work is dedicated to Sir Henry Halford, Bart. the dignified President of the Royal College of Physicians, and contains the result of* fifty-six years' *practice. This is no trifling recommendation.*

---

19. A Series of Questions and Answers, for the Use of Gentlemen preparing for their examination at Apothecaries' Hall; with copious and useful Tables annexed. By CHARLES MINGAY SYDER, Licentiate of the Honorable Society of Apothecaries, &c. Duodecimo, 126 pages. London, 1821.

☞ *This is the only work which we have seen for the purpose of preparing gentlemen for Apothecaries' Hall, in the shape of Questions and Answers. We look upon it as very well adapted to the object designed.*

---

20. A Treatise on the Mineral Water of ASKERN, including a Description of the Village, History of the Spaw, &c. By T. LE GAY BREWERTON, Licentiate of the Royal College of Surgeons, and Fellow of the Royal Physical Society, Edinburgh. One vol. 8vo, pp. 144.

☞ *Some account of this little volume shortly.*

---

21. The Scourge of Venus and Mercury, represented in a Treatise of the Venereal Disease; giving a succinct account of the Nature, Causes, and Symptoms of that dreadful Distemper; *and the fatal consequences arising from Mercurial Cures.* Unto which is added, the *true way* of curing (without a particle of mercury) not only the *consummate* and *inveterate,* but also the *mercurial Pox*; found to be more dangerous than the Pox itself. By S. SINTELAER, Practitioner in Physic. Octavo, pp. 363. London, 1709.

☞ *We return Dr. Dougal Campbell our thanks for this curious volume, containing most of the* MODERN *mercurial and anti-mercurial discoveries. We hope to give some amusing extracts from it in our next number.*

---

22. Observations on Syphilis, principally with reference to the use of Mercury in that Disease. By JOHN BACOT, Member of the Royal College of Surgeons, and late Surgeon of the Grenadier Regiment of Guards. Octavo, pp. 115. London, 1821.

23. Essays on the Female Economy. On the periodical Discharge of the Human Female; with new Views of its Nature, Causes, and Influence on Disease; to which are added, Directions for its Management in the different Stages of Life, &c. &c. By John Power, M. D. Physician-Accoucheur to the Westminster Lying-in Institution, &c. One vol. 8vo, pp. 101, with a plate. London, 1821.

---

24. A Treatise on Acupuncturation; being a Description of a Surgical Operation originally peculiar to the Japanese and Chinese, and by them denominated ZIN KING, now introduced into European practice, with directions for its performance, and Cases illustrating its success. By JAMES MORSS CHURCHILL, Member of the Royal College of Surgeons in London. Octavo, pp. 86, with a plate of the instrument. London, 1821.

---

25. The Principles of Forensic Medicine, systematically arranged, and applied to British practice. By JOHN GORDON SMITH, M. D. Octavo, pp. 503. London, 1821.

---

26. An Essay on the Disorders and Treatment of the Teeth. By ELEAZAR PARMLY, Dentist. Small octavo, pp 62. London, 1821.

☞ *The author of this little volume, and his brother now in America, we know to be able and ingenious dentists. The present little volume contains perspicuous Directions for the Management of the Teeth, and sensible Observations on the Causes of their Decay.*

---

27. The Philadelphia Journal of the Medical and Physical Sciences. Edited by N. CHAPMAN, M. D. No. 3, May, 1821. Octavo, pp. 208.

☞ *In acknowledging the receipt of the above, we beg to inform Dr. Chapman that the Medico-Chirurgical Review shall be regularly forwarded in return. The fifth number, being the first of a volume, was transmitted by the hands of Professor Caldwell, last month.*

---

28. A Practical Treatise on Ringworm of the Scalp, Scalled Head, and the other Species of Porrigo. By SAMUEL PLUMBE, Member of the Royal College of Surgeons of London, &c. Octavo, pp. 104, with a plate. London, 1821.

---

29. The Physician's Guide; being a popular Dissertation on Fevers, Inflammations, and all diseases connected with them; comprising Observations on the Use and Abuse of Bloodletting, Mercury, Cathartics, Stimulants, Diet, &c. &c. By ADAM DODS, M. D. Octavo, pp. 320. Worcester, 1821.

---

30. Examen des Doctrines Medicales et des Systemes de Nosologie; ouvrage dans lequel se trouve fondu l'examen de la doctrine medicale generalement adoptée, &c, precédé de propositions renferment la substance de la Medecine physiologique. Par F. I. V. BROUSSAIS, &c. &c. Two vol. 8vo, pp. 874. Paris, 1821. Transmitted through Dr. Ducamp.

---

31. Memoir au Roi et son conseil des Ministres, et aux Chambres; ou protestation contre le Travail de la Commission Sanitaire, &c. Par M. JEAN DEVEZE, M. D. &c. &c. Quarto, pp. 33. Paris, 1821.

---

32. Analyse Chimique des Quinquina. Par M. M. PELLETIER, et CAVENTON, Suivie d'Observations Medicales sur l'Emploi de la Quinine et de la Cinchonine. Octavo, pp. 88. Paris, 1821.

---

33. Observations on the Nature and Treatment of the Fever prevailing in London, &c. By ARCHIBALD M'DONNEL, Member of the Royal College of Surgeons, in London. Octavo, pp. 28. London, 1818.

34. A Treatise on the Anatomy and Physiology of the Mucous Membranes; with illustrative Pathological Observations. From the French of Xavier Bichat. By Joseph Houlton, Member of the Royal College of Surgeons in London. Octavo, pp. 100. London, 1821.

☞ *We are happy to see this little Treatise of the illustrious Bichat in an English dress. It well deserves, and will well repay, an attentive study of its important contents.*

---

35. Medical Dissertations on Hæmoptysis, or the Spitting of Blood, and on Suppuration; which obtained the Roylston Premiums for the years 1818 and 1820. By John Ware, M. D. Fellow of the Massachusets Medical Society. Octavo, pp. 95. Boston, 1820.

☞ *We return Dr. Ware our thanks for this little volume, which shall have due notice as soon as possible.*

---

36. Elements of Medical Logic, illustrated by Practical Proofs and Examples. Second edition, with large additions, particularly in the practical part. By Sir Gilbert Blane, Bart. &c. &c. Physician to the King. Octavo, pp. 280. London, August, 1821.

☞ *The First edition was reviewed in a former number of this Journal. The favourable reception which it has met with from the medical world, has induced this able and excellent author, as well from a sense of gratitude as duty, to use his best endeavours to render the present impression still more worthy of their acceptance. The work, as may readily be conceived, bears the stamp of profound thought, extensive erudition, and sound judgment. May this amiable and talented physician live to see many editions issue from the press, to roll down his name on the stream of time, as one of those who shone illustrious in the early part of the nineteenth century.*

---

37. An Essay on the Medical Application of Electricity and Galvanism; with a concise descriptive Account of Disease. By James Price, Surgeon, Member of the Royal College of Surgeons, late in His Majesty's Service, &c. One vol. 8vo, pp. 142. London, 1821.

---

38. Observations on certain Affections of the Head, commonly called Head-aches; with a view to their more complete Elucidation, Prevention, and Cure. Together with some brief Remarks on Digestion and Indigestion. By James Farmer, Member of the Royal College of Surgeons in London, and Licentiate of Midwifery of the Royal College of Physicians of Dublin. Duodecimo, pp. 88. London, 1821.

---

39. A Treatise on Burns and Scalds: detailing the best methods of treating those accidents, as practised in the London Hospitals, and by the most celebrated medical practitioners. By John Augustine Waller, Surgeon, R. N. Octodecimo, pp. 61. London, 1821.

☞ *This little miniature volume presents portraits of, and some criticisms on, the Plans of Cleghorn, Earl, Kentish, &c.*

---

40. Remarks on the Epidemic Yellow Fever, which has appeared, at intervals, on the South Coasts of Spain, since the year 1800. By Robert Jackson, M. D. One vol. 8vo, pp. 207, 8s. boards. London, August, 1821.

☞ *This interesting little work is the result of the veteran, able, and zealous Dr. Jackson's personal researches, on the coasts alluded to. Only* 250 *copies are printed, and those at the author's own expense. We are*

*sure our brethren will be eager to peruse this (perhaps last!) production from the pen of an indefatigable promoter of medical knowledge; and we can with great safety assert that the intrinsic merit of the work is such as to render it a rich treat to the profession. We shall shew proof of this in our next number.*

---

41. Researches into the Laws and Phenomena of Pestilence; including a Medical Sketch and Review of the Plague of London, in 1665; and Remarks on Quarantine. With an Appendix, containing Extracts and Observations relative to the Plagues of Morocco, Malta, Noya, and Corfu; being the subject of the Anniversary Oration, delivered before the Medical Society of London, in the Spring of 1820, and published at their request. By THOMAS HANCOCK, M. D. Licentiate of the Royal College of Physicians, and Physician to the City and Finsbury Dispensaries. Octavo, pp. 378. London, July 1821.

---

42. On the Nature, Symptoms, and Treatment, of the different Species of Amaurosis, or Gutta Serena; illustrated by Cases. By JOHN STEVENSON, Esq. Surgeon-Oculist and Aurist to His Royal Highness the Duke of York, and His Royal Highness Prince Leopold of Saxe Cobourg; and Member of the Royal College of Surgeons, &c. &c. One vol. 8vo, London, 1821.

---

43. A Manual of Logic, in which the Art is rendered practical and useful upon a Principle entirely new: illustrated with sensible Figures of every Species of Proposition, and every Form of Syllogism. By Mr. J. W. CARVILL, Lecturer on Natural Philosophy, &c. One small volume, duodecimo, pp. 75. London, 1821.

☞ *Mr. C. is a medical practitioner, and this little manual appears to us to be an ingenious performance.*

---

The Extra-Limites department of this Journal having now been open nearly twelve months without any of the numerous authors, whose works were reviewed, having taken advantage of it to defend themselves, the fair inference is, that the conduct pursued by the reviewers has been generally approved. Nevertheless, a few remonstrances have been *privately* addressed to the Editor, which he has not answered, because he conceives that, in laying open a door for public redress, he is not bound to have his time intruded on, or his feelings harrassed by private appeals. He considers himself as in no way responsible for individual criticisms in the Journal, but only as far as respects propriety of language, expressions, and general conduct.

### TO CORRESPONDENTS.

Dr. Crane's communication on *Bulimia Emetica* is received.

JUSTITIA is respectfully informed that we can never interfere with the conduct of other journalists towards authors or writers of papers in periodical works. The *Extra-Limites* department is open to them to defend themselves, *in propria persona.*

AMICUS'S advice is friendly, but we cannot follow it. It would be very unwise in us to notice the scurrilities that may be lavished on us by men who hope to gain notoriety by these attacks. Next to the approbation of the *good,* we prize the obloquy of the *unprincipled* portion of society. The virulence of the *latter* rises, of course, in exact proportion and *pari passu* with our success. Let Amicus remember BRAMBLE'S answer to an extorter of money, who held a lampoon in one hand and a panegyric in the other—"Publish your lampoon whenever you please; but if you dare to publish a panegyric of me, I'll break every bone in your skin." These are exactly our sentiments.

"Hated by knaves—and knaves to hate—
"Be this our maxim—this our fate."

Criticus has our thanks for his hints and communications. Some of the latter he will see in the proper place. We wish to know his real name and address.

Senex's information will be turned to good account when the work comes under review. We shall always be obliged to him, or any other one, who will favour us with remarks on points of doctrine or practice, contained in the publicati..ns of the day. If considered disinterested and judicious, they will be applied in the right quarter.

The favours of Dr. O.—, of M.R.—, of G.—, and T. S. came safe to hand.

### *Additional Subscribers since June* 1*st*, 1821.

Allen, Oswald, Esq. York.
Asled, Mr. Surgeon, Halifax
Burder, Dr. King's-road Bedford Row
Baker, William, Esq. Charlotte-street, Bedford-square
Brown, Mr. Surgeon, Brighton
Bailey, Mr. Charles, Kirkleatham, Yorkshire
Bailey, Mr. H. W. Thetford
Bridger, Sam. M.D. Honorable Company's Service

Cooper, Mr. C. Surgeon, Tooting
Campbell, Dr. Dougall, Surgeon, Royal Artillery, member of the Royal Physical Society of Edinburgh, at present resident Physician (by permission of the King of France) at Boulogne, Sur Mer
Coyne, Doctor, Sligo, Ireland.
Duncan, Mr. Surgeon, Wrotham
Deal Medical Society
Doak, Michael, Esq. Surg. R. N.
Darling, Dr. Brunswick-square
Dingle, Mr. Bookseller, Bury, six copies
Davies, Mr. T. L. O.
Erskine, William, Esq. Bombay Establishment
Eachus, Mr. George, Surgeon, Saffron Walden, Essex
Ferguson, Hugh, Esq. Surg. R.N.
Foulkes, Ed. Esq. Surg. Ruyton, (Eleven Towns) Salop
Fraser, Mr. A. Doughty-street
Faculty of Physicians and Surgeons, Glasgow
Fitzgerald, Mr. Percy-street
Gilbert, Mr. J.B. Surgeon, Chapel Row, Worthing
Howel, Mr. J. Surg. Wandsworth
Hulke, William, Esq. Deal
Huggins, Mr. Surgeon, Derby
Hill, Mr. Ninian, Greenock
Johnstone, J. C. Esq. Memb. R. Col. Surg. Lond. His Majesty's Ship Requin, Grenada, W. Ind.
Jefferson, Mr. T. R. Surg. Middlesex-place, New Road
Kinderwood, Mr. Surgeon-Accoucheur to the Manchester Lying-in-Hospital
Langstaff, Mr. W. H. Surgeon, Stockton
Lara, Benj. M. D. of the Royal College of Physicians, Ed. Resident Physician, Portsea, Hants
Loxley, G. Esq. Member of the Royal Coll. Surg. Rotherane
M'Millan, Dr. Deputy Inspector Army Hospitals
Major, T. Esq. Surg. Hungerford, Berks, Memb. Royal College of Surg. and Society of Apoth. and late Surg. in H.M. Navy.
Mauigy, Daniel Pallot, Esq. Guernsey
Macdowal, Mr. Surgeon, Bengal
Morton, Mr. Chas. Londonderry
M'Corkell, Isaac. Esq. Surgeon, Jamaica
Mitchell, Mr. Thomas, Member of Royal College of Surgeons, and Licentiate of the Apoth. Comp. Whitehaven, Cumberland
Maxton, Mr. James, Surg. Crief
Pitt, Mr. Surgeon, Brighton
Paynter, J. W. Esq. Surgeon, Pembroke
Parkin, Henry, M. D. Cawsand, Plymouth Dock
Pedder, George, Esq. Member of the Royal College of Surgeons, Ryde, Isle of Wight.
Poysers, Mr. Surg. Werkworth
Quead, Mr. Surgeon, Royal Navy
Ramadge, Dr. Thavies Inn, Holborn
Rogers, P. Esq. Member of the Royal Coll. of Surg. Cornwall
Rocliffe, Dr. W. L. Easingwood, Yorkshire
Roberts, Mr. John, Surgeon, Lothbury.
Scott, P. N. Esq. Surg. Norwich
Simpson, Thomas, Esq. Surgeon, Knaresbro'
Simpson, Dr. Malton, Yorkshire
Sandwich Medical Society
Sprague, Mr. Surgeon, Kingston-upon-Thames
Syder, Mr. C. M. Surgeon, Lancaster Street, Burton Crescent
Stevenson, John, Esq. Great Russel-Street
Stephenson, Surgeon, 48th Regt.
Smith, Southwood, M.D. Licentiate of the Royal Coll. of Physicians, London, Physician to the London Dispensary, Trinity Square
Thomson, John, M. D. Edinburgh
Taylor, Mr. Surgeon, Steyne, Brighton
Thomas, Mr. Isle of France
Watson, Mr. (Student) Edinburgh
Young, Mr. Ploughman
York County Hospital Library

